# Impact of Molecular Modifications on the Immunogenicity and Efficacy of Recombinant Raccoon Poxvirus-Vectored Rabies Vaccine Candidates in Mice

**DOI:** 10.3390/vaccines9121436

**Published:** 2021-12-04

**Authors:** Carly M. Malavé, Jaime Lopera-Madrid, Lex G. Medina-Magües, Tonie E. Rocke, Jorge E. Osorio

**Affiliations:** 1 U.S. Geological Survey, National Wildlife Health Center, Madison, WI 53711, USA; cmalave@usgs.gov; 2 Department of Pathobiological Sciences, School of Veterinary Medicine, University of Wisconsin-Madison, Madison, WI 53706, USA; loperamadrid@wisc.edu (J.L.-M.); medinamagues@wisc.edu (L.G.M.-M.)

**Keywords:** rabies, rabies virus, RABV, raccoon poxvirus, recombinant, vaccine, A/J mice

## Abstract

Rabies is an ancient disease that is responsible for approximately 59,000 human deaths annually. Bats (Order *Chiroptera*) are thought to be the original hosts of rabies virus (RABV) and currently account for most rabies cases in wildlife in the Americas. Vaccination is being used to manage rabies in other wildlife reservoirs like fox and raccoon, but no rabies vaccine is available for bats. We previously developed a recombinant raccoonpox virus (RCN) vaccine candidate expressing a mosaic glycoprotein (MoG) gene that protected mice and big brown bats when challenged with RABV. In this study, we developed two new recombinant RCN candidates expressing MoG (RCN-tPA-MoG and RCN-SS-TD-MoG) with the aim of improving RCN-MoG. We assessed and compared in vitro expression, in vivo immunogenicity, and protective efficacy in vaccinated mice challenged intracerebrally with RABV. All three candidates induced significant humoral immune responses, and inoculation with RCN-tPA-MoG or RCN-MoG significantly increased survival after RABV challenge. These results demonstrate the importance of considering molecular elements in the design of vaccines, and that vaccination with either RCN-tPA-MoG or RCN-MoG confers adequate protection from rabies infection, and either may be a sufficient vaccine candidate for bats in future work.

## 1. Introduction

Rabies is a devastating neurological disease that afflicts humans, domestic animals, and wildlife, and is caused by rabies virus (RABV), a negative strand lyssavirus of the family *Rhabdoviridae*. If untreated, infection with RABV will result in fatal encephalitis. The primary source of rabies cases in humans are dog bites, and approximately 40% of people bitten by rabid animals are children under 15 [1]. Pre- and post-exposure prophylaxis are available in the form of a human rabies vaccine and human rabies immune globulin, but these treatments are expensive and not readily available to populations that need them most. Rabies prevention efforts cost approximately $300 million annually in the United States and over $1 billion globally [2]. 

In 2017, 91% of all reported rabies cases in animals in the United States came from wildlife. Over 30% of those cases occurred in bats, and the number of cases per year has been on the rise since the 1990s [3]. In Central and South America, vampire bat rabies is responsible for the deaths of thousands of livestock and hundreds of human rabies cases annually [4,5,6]. Methods to mitigate bat rabies largely rely on depopulation, but this approach has not been very effective, as depopulation often results in dispersal and further spread of disease [7,8]. In contrast, current rabies management strategies for wild carnivores have been centered on oral rabies vaccination (ORV) programs using RABORAL VR-G^®^, a recombinant vaccinia virus vectored vaccine expressing the envelope glycoprotein G. Distribution of baits containing RABORAL VR-G^®^ has successfully reduced rabies cases in several terrestrial carnivore species in North America and Europe, including wild raccoons, gray foxes, and coyotes [9,10]. Although VR-G was previously tested in captive vampire bats and conferred protection in up to 60% of vaccinated bats [11], it has not been field tested or applied to wild bats. The viral vector, vaccinia, causes disease in humans and cattle, raising questions about its safety profile for use in wild populations and vampire bats, in particular, due to their blood feeding habits [12,13]. Additionally, bats carry several antigenic variants of RABV and other phylogroup 1 (PG-1) lyssaviruses that cause rabies-like disease, precipitating the need for a vaccine with broader antigenic coverage [14,15,16].

Previously, our research group developed a recombinant raccoonpox virus (RCN) vaccine candidate that expressed a mosaic glycoprotein (MoG) gene. RCN-MoG is a broad-spectrum vaccine designed to provide protection across all lyssaviruses in PG-1 and has a demonstrated 61% antigenic coverage across 664 G sequences in PG-1 [17]. Inoculation with RCN-MoG conferred protection in mice and big brown bats (*Eptesicus fuscus*) when challenged with RABV, and in vitro assays revealed mosaic glycoprotein expression in cell culture [17]. Interestingly, some bats that received RCN-MoG topically or oronasally did not seroconvert after vaccination (despite surviving challenge), and in vitro assays of the RCN-MoG construct yielded relatively low quantities of MoG protein.

In order to improve in vitro expression and immunogenicity in vivo, we modified the MoG gene cassette to create new second-generation recombinant vaccines. In this study, we aimed to enhance MoG expression through several molecular modifications of the recombinant RCN-MoG construct and to improve antibody production and vaccine efficacy in the mouse model. Specifically, RCN-tPA-MoG was developed by adding the tissue plasminogen activator (tPA) secretory signal and the strong PrH5m promoter to enhance MoG gene expression. RCN-SS-TD-MoG was developed by adding the human IgG signal peptide (SS) and modifying the transmembrane domain (TD) sequence of the MoG gene to be homologous with the RABV CVS-11 strain; SS was added to increase protein expression and the TD was modified to ensure correct G protein conformation. In vitro expression, immunogenicity, and efficacy of all three constructs were compared through serological assays and a rabies challenge study. We demonstrated that RCN-tPA-MoG and RCN-SS-TD-MoG could induce strong humoral immune responses in mice, similar to that of RCN-MoG. Additionally, vaccination with either RCN-tPA-MoG or RCN-MoG had a positive effect on survival against RABV.

## 2. Materials and Methods

### 2.1. Cells and Viruses

Recombinant viruses, RCN-tPA-MoG and RCN-SS-TD-MoG, were produced and amplified on cell monolayers of human embryonic kidney cells (HEK-293, ATCC #CRL-1573) or African Green monkey (*Cercopithecus aethiops*) kidney epithelial cells (Vero, ATCC #CCL-18). Cell cultures were maintained at 37 °C with 5% CO_2_ in Dulbecco’s Modified Eagle Medium (DMEM) supplemented with 1–5% fetal bovine serum (FBS). Recombinant RCN-MoG and RCN-GFP used in this study are previously described [17,18]. RABV CVS-11 (Genbank accession #AB069973) used in the mouse challenge and for the rabies antibody testing was provided by the Centers for Disease Control.

Baby hamster (*Mesocricetus auratus*) kidney cells (BHK-21, ATCC #CCL-10) were used for rabies serology and maintained at 37 °C with 5% CO_2_ in minimum essential media supplemented with 10% FBS (MEM-10).

### 2.2. Design and Construction of Recombinant Viruses

**Figure 1:**
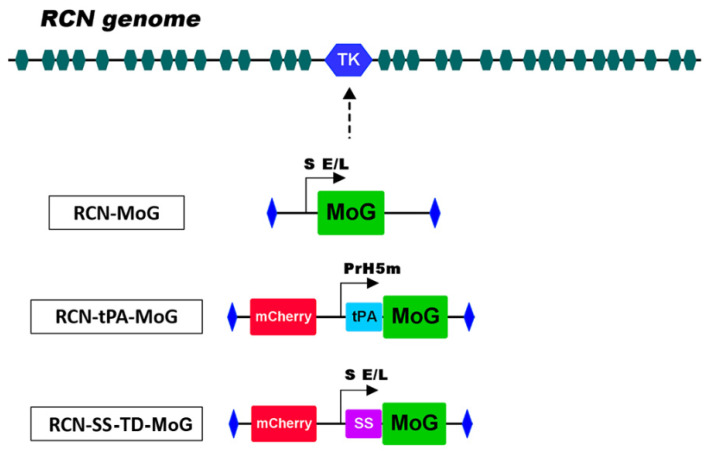
A map of the RCN genome showing insertion of recombinant cassettes at the *tk* site. The RCN-MoG cassette contains the mosaic glycoprotein (**MoG**) gene under the control of the S E/L promotor. The RCN-tPA-MoG cassette map contains the mCherry fluorescent marker, the tissue plasminogen activator (**tPA**) secretory signal under the control of the PrH5m promoter, and the MoG gene. The SS-TD-MoG cassette contains the mCherry fluorescent marker, the human IgG secretory signal (**SS**) under the control of the S E/L promoter, and the MoG gene with the CVS-11 transmembrane domain sequence. The secretory signals (**tPA** and **SS**) and promoters (**S E/L** and **PrH5m**) were added to their respective cassettes to enhance expression and extracellular secretion of MoG.

The design and *in silico* assessment of the mosaic rabies glycoprotein (MoG) is described elsewhere [17]. The MoG gene cassette utilized in recombinant RCN-MoG was modified to generate the tPA-MoG and SS-TD-MoG cassettes. For tPA-MoG, the first 19 amino acids (aa) of MoG were replaced by secretory signal of the tissue plasminogen activator (tPA, aa 1-22, L00141). Also, the strong PrH5m promoter [19] was used to drive the expression of the MoG gene (Figure 1). The modifications for the SS-TD-MoG cassette include replacement of the first 19 aa of MoG by the human IgG signal peptide (amino acid sequence MELGLSWVFLVAILEGVQCE), and the alteration of the MoG gene TD (Figure 1). The TD of the original MoG sequence has approximately 50% aa homology to the G gene of RABV CVS-11; therefore, the TD region of MoG was modified to have 100% homology with the CVS-11 sequence to ensure proper conformation. The strong synthetic early/late (S E/L) promoter used in RCN-MoG was also included to direct expression of MoG.

DNA cassettes containing the sequences for tPA-MoG and SS-TD-MoG, as well as the mCherry gene under the control of a late p11 promoter, and flanking sequences from the RCN thymidine kinase (*tk*) gene were synthesized. These cassettes were inserted into a wild-type RCN (RCN-*tk*-GFP), in which the *tk* gene was replaced with the green fluorescent protein (GFP) [20]. The addition and subsequent expression of the mCherry protein allows for visual-based selection and permits an easy distinction between recombinant (red) and wild-type (green) viruses. The tPA-MoG and SS-TD-MoG plasmids were commercially generated (GenScript, Nanjing, China) and co-transfected into HEK cells infected with RCN-GFP at a multiplicity of infection (MOI) of 0.05 using the FuGENE^®^ HD transfection reagent (Promega, Fitchburg, WI, USA). After expansion, successful insertion was confirmed through DNA extraction of the recombinant viruses using a *Quick*-DNA Miniprep extraction kit (Zymo Research, Irvine, CA, USA) and PCR amplification of the cassettes (2350 bp). Recombinant viruses were then amplified and purified for in vivo use. Briefly, Vero cells in T-175 flasks were infected with each virus at an MOI of 0.1 and collected after 24 h. Viruses were centrifuged, and pellets were collected in Tris-HCl then purified via ultracentrifugation through a Tris-HCl/36% sucrose gradient. DNA sequences of the stock viruses were confirmed through Sanger sequencing (University of Wisconsin-Madison Biotechnology Center, Madison, WI, USA).

### 2.3. Immunofluorescence Assay for In-Vitro Expression

Vero cells in 6-well plates were infected at an MOI of 0.5 with RCN-MoG, RCN-tPA-MoG, RCN-SS-TD-MoG, and RCN-GFP; an additional well was left uninfected to serve as a negative infection control. After 24 h, cells were fixed with a 1:1 mix of 100% methanol and 100% acetone, washed with phosphate-buffered saline (PBS) and permeabilized with a PBS/0.25% Triton X-100 solution for 15 min. The plates were then rinsed and blocked with PBS/3% bovine serum albumin (BSA) solution for 1 h, and after blocking, plates were incubated with a mouse anti-rabies glycoprotein antibody (Abcam, Cambridge, UK). After 2 h, the wells were washed three times for 10 min each with a PBS/1.5% BSA/0.05% Triton X-100 washing solution. A secondary fluorescent antibody with a 1:2000 dilution of Alexa Flour 594 tagged goat anti-mouse antibody (Invitrogen, Thermo Fisher Scientific Inc., Waltham, MA, USA) was added to each well and incubated for 1 h at room temperature. After washing, plates were visualized under a fluorescent microscope (excitation wavelength 590 nm, emission wavelength 617 nm; AMG EVOSfl, Thermo Fisher Scientific Inc., Waltham, MA, USA).

### 2.4. Quantification of Expression in Immunofluorescence Assay

Images taken from the immunofluorescence assay were quantified using an image processing protocol described in Meza et al. [21]. Briefly, plates were photographed at 20× magnification (70% brightness) under a fluorescent microscope (excitation wavelength 590 nm, emission wavelength 617 nm; AMG EVOSfl, Thermo Fisher Scientific Inc., Waltham, MA, USA). Three equally sized fields from the center of each well were selected and photographed (left to right). Images were processed through ImageJ (version 2.1.0/1.352k) and Fiji [22,23]. First, the images were transformed into an 8-bit binary representation (every pixel stored as a single bit), indicating the presence (white) and absence (black in the background) of GFP fluorescence. Then, images were processed using the Autolocal Threshold Phansalkar plugin (size radius 1–5), and the function “Analyze Particles” was used to count the total number of fluorescent particles per field (i.e., cells expressing MoG protein). This command grouped and counted the white pixels with a general size area and circularity to capture every area of fluorescence (size area: 0–infinity; circularity: 1). A summary including the number of white particles counted in each image was generated and used in the analysis.

### 2.5. Virus Growth Curve

Vero cells in 6-well plates were infected at an MOI of 0.5 with RCN-MoG, RCN-tPA-MoG, RCN-SS-TD-MoG, and RCN-GFP; additional wells were left uninfected to serve as a negative infection control. Cells were incubated at 37 °C and harvested at 12, 24, 36, and 48 h post-inoculation. Virus titration was performed in 96-well plates using Vero cells seeded at a concentration of 10^4^ cells/well to achieve confluency after 24 h incubation at 37 °C. Plates were infected with 50 μL of a 10-fold serial dilution of recombinant virus (10^−1^ to 10^−12^) with 8 replicates per dilution and incubated for 3 days. Plates were visualized under a fluorescent microscope, where wells displaying one or more viral plaques were designated as positive. Virus titers were calculated using the Reed-Muench method [24].

### 2.6. Immunogenicity and Challenge Study

Forty female A/J mice (3-week-old) were purchased from Jackson Laboratory (JAX, Sacramento, CA, USA) and were housed in the vivarium of the Hanson Biomedical Sciences Building (University of Wisconsin-Madison, Madison, WI, USA). Laboratory mice are the standard animal model for rabies vaccine and viral challenge studies. Mice can display clinical signs of rabies infection and can develop robust humoral immune responses from vaccination. Mice are also much easier to obtain and house than wild bats, the target species in this case, and, thus, they are useful for preliminary assessments of vaccines. After a 48-h acclimation period, mice were separated into four treatment groups, with two cages per group and five mice per cage. Each treatment group (*n* = 10) was inoculated via intramuscular injection (thigh) with 1 × 10^7^ pfu in 50 µL of RCN-MoG, RCN-tPA-MoG, RCN-SS-TD-MoG, and PBS. Intramuscular injection was selected as the delivery method in lieu of oral vaccination, as oral replication of RCN has not yet been evaluated in mice. Blood was collected via maxillary lance at 14 days post vaccination (dpv), 27 dpv (1 day before RABV challenge), and time of death or the end of the study for surviving mice. Serum was aliquoted, stored at −80 °C, and later heat-inactivated for 30 min at 56 °C before serological analysis. At 28 dpv, all mice were challenged with 8.8 × 10^3^ pfu of CVS-11 RABV in 30 µL via intracerebral injection and monitored for 2 weeks. Mice were weighed daily, monitored twice daily, and were euthanized if they had lost more than 20% of their body weight and/or if they presented with clinical rabies signs for two consecutive visits.

### 2.7. Rabies Diagnosis and Serology

Serum samples were analyzed for detectable rabies virus neutralizing antibody (RVNA) titers using a modified micro neutralization assay [25], based on the Rapid Fluorescent Focus Inhibition Test [26]. Briefly, mouse sera were mixed with BHK-21 cells and CVS-11 RABV in MEM-10 media in a 4-well Teflon coated slide; after incubation, slides were fixed with acetone, stained with a FITC RABV stain (Fujirebio U.S. Inc., Malvern, PA, USA), and visualized under a fluorescent microscope. Ten microscopic fields per well were read for presence and absence of fluorescing cells, and the number of fluorescent fields per well were used to calculate the endpoint titers via the Reed-Muench method [24]. Titers were converted to international units per milliliter (IU/mL) by comparison to a standard rabies immunoglobulin (SRIG) positive control with 2 IU/mL. For the objective of this study, the positive cutoff value (greater than or equal to 0.5 IU/mL) was determined by at least 50% neutralization of the CVS-11 challenge virus (50 focus forming doses) in a 1:10 dilution of the SRIG. Mouse brains were assessed for rabies infection using the direct fluorescent antibody test (DFA). After brain impressions were fixed in acetone, slides were stained with a FITC-labelled monoclonal antibody (mAB) conjugate (Fujirebio U.S. Inc., Malvern, PA, USA) and visualized under a fluorescent microscope, as described elsewhere [27].

### 2.8. Statistical Analysis

The Kruskal-Wallis test was used to analyze neutralizing antibody titers between groups of mice, and the Mann-Whitney test was used to compare two treatment groups within a time point. The Kruskal-Wallis test was also used to compare numbers of particles counted in the images of the immunofluorescence assays. Kaplan Meier survival analyses were performed to compare survival between vaccinated mice and control mice. Probability values (P) of less than or equal to 0.05 were considered significant. GraphPad Prism (v8.2.1) software (San Diego, CA, USA) was used for all statistical analyses.

## 3. Results

### 3.1. In-Vitro Protein Expression and Viral Growth

**Figure 2:**
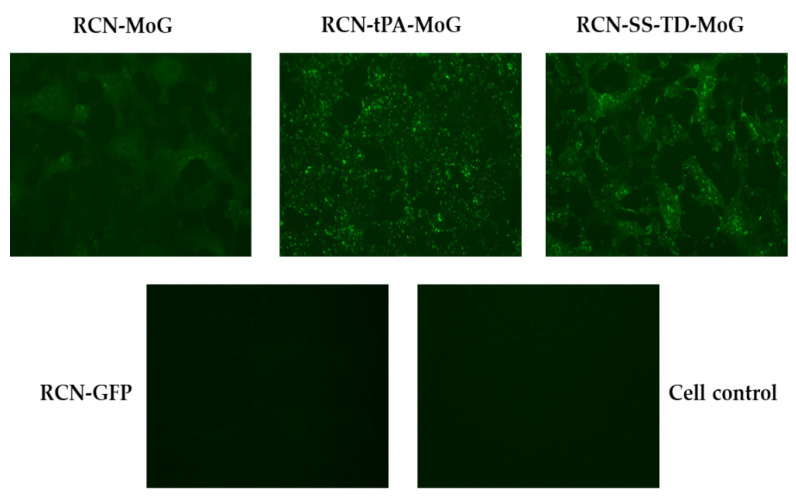
Immunofluorescence of raccoonpox constructs RCN-MoG, RCN-tPA-MoG, and RCN-SS-TD-MoG expressing mosaic lyssavirus phylogroup I glycoprotein (MoG) 24 h after infection. RCN expressing green fluorescent protein (GFP) and a well of uninfected cells (Cell control) were used as negative controls.

Immunofluorescence assays confirmed the presence of RABV glycoprotein antigen in RCN-tPA-MoG, RCN-SS-TD-MoG, and RCN-MoG when compared to RCN-GFP and the cell negative control (Figure 2).

**Figure 3:**
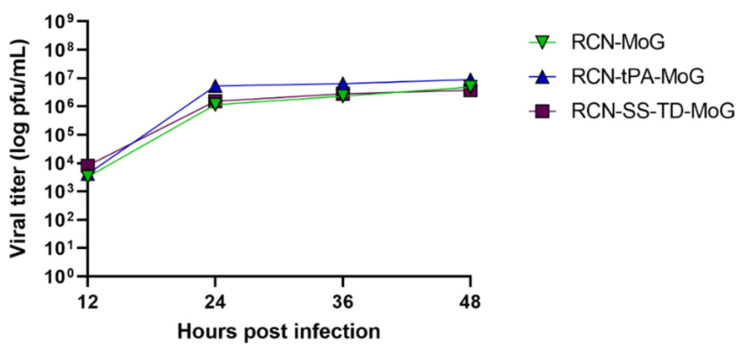
One-step growth curves for recombinant RCN constructs. Vero cells were infected with RCN-MoG, RCN-tPA-MoG, and RCN-SS-TD-MoG, collected at the indicated time points, and quantified with a plaque assay.

Cells infected with any of the three constructs appeared to have similar concentrations of rabies antigen. However, quantification of the immunofluorescence images yielded statistically significant differences between the three viruses (*p* = 0.004). More specifically, RCN-tPA-MoG produced the highest mean of fluorescent particles, followed by RCN-SS-TD-MoG, then RCN-MoG (Table 1). The results of the viral growth assays show similar growth kinetics between the three constructs (Figure 3).

### 3.2. Mouse Immunogenicity and Challenge Study

**Figure 4:**
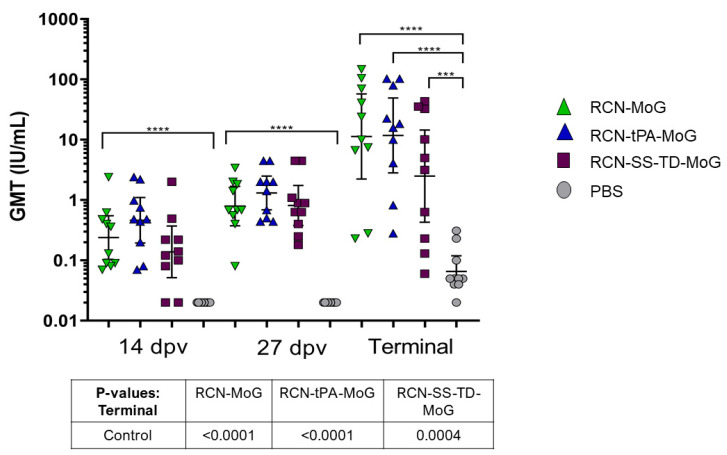
Geometric mean serum titers of rabies neutralizing antibodies (IU/mL) in mice 14 dpv, 27 dpv, and at the end of the study. Mice were inoculated with either RCN-MoG, RCN-tPA-MoG, RCN-SS-TD-MoG, or PBS. No significant differences were observed between vaccinated groups at any time point (*p* > 0.05). Significant differences were detected between vaccinated and control groups at each time point *(p* ≤ 0.0001, except for RCN-SS-TD-MoG group at end of study where *p* = 0.0004). Asterisks on the graph indicate statistical significance (i.e., *** = *p* ≤ 0.001 and **** = *p* ≤ 0.0001).

Mice that received any vaccine construct had significantly higher titers of RVNAs compared to the control group at each time point (Figure 4). We did not detect statistically significant differences in RVNA titers between the three vaccinated groups at days 14, 27, and at the end of the study.

**Figure 5:**
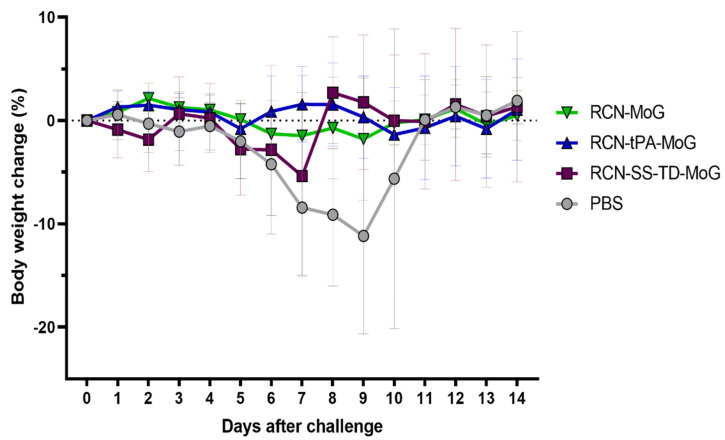
Changes in mean mouse body weights (as percentage of weight change from day 0) over time (in days) following challenge with RABV. Weight change is presented as percentage of initial body weight lost or gained after challenge, with standard deviations. The percent change in mean weights of mice in the control group (PBS) trends downward from day 6 to day 9.

**Figure 6:**
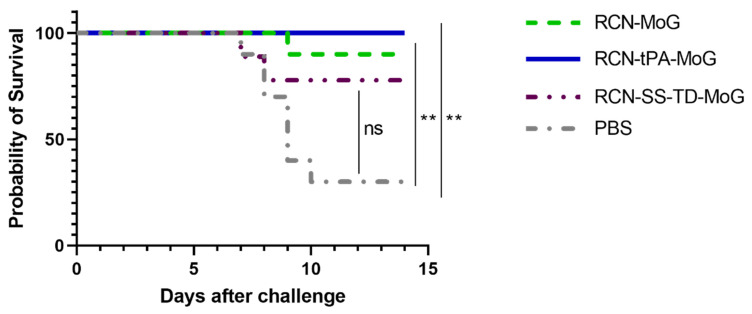
Efficacies of RCN-vectored rabies vaccines in mice after intracerebral challenge with the CVS-11 strain of rabies virus. The RCN-tPA-MoG group had 100% survival from challenge, compared to 90% survival in the RCN-MoG group, 78% in RCN-SS-TD-MoG, and 30% survival in the PBS group. Survival in mice vaccinated with RCN-tPA-MoG and RCN-MoG was significantly higher (*p* = 0.0013, *p* = 0.0061, respectively) than negative controls, but there were no significant differences between vaccine groups and a marginally insignificant difference between the RCN-SS-TD-MoG and control groups (*p* = 0.0503). Asterisks on the graph indicate statistical significance (i.e., ** = *p* ≤ 0.01).

One mouse in the RCN-SS-TD-MoG group died before challenge due to non-study related complications. Weights after RABV challenge were similar between all groups until the mean weight change of the control group began to decline at day 6 and rose again at day 10 following euthanasia or death of sick mice in that group (Figure 5). All (100%) of the mice in the RCN-tPA-MoG group survived challenge, followed by 90% (9/10) of the RCN-MoG group, 78% (7/9) of the RCN-SS-TD-MoG group, and 30% (3/10) of the PBS control group (Figure 6). No significant differences were detected between the survival rates of the vaccinated groups, but the RCN-tPA-MoG and RCN-MoG vaccinated mice had significantly higher survivorship than the control group (*p* = 0.0013 and 0.0061, respectively). Vaccination with RCN-SS-TD-MoG was marginally insignificant compared to controls (*p* = 0.0503). All mice that were euthanized during challenge with suspected rabies were confirmed positive by DFA.

## 4. Discussion

Bats (Order *Chiroptera*) have consistently played a substantial role in the transmission of RABV to humans and domestic animals, yet an efficacious rabies vaccine specifically designed for bats remains a challenge. In this study, we demonstrated that vaccination of mice with second-generation recombinant rabies vaccines, RCN-tPA-MoG and RCN-SS-TD-MoG, elicited RVNA titers comparable to the original vaccine construct, RCN-MoG. Although no differences in titers were detected among vaccine-treated groups after challenge with RABV CVS-11, those that received either RCN-tPA-MoG or RCN-MoG survived at higher rates (100% and 90% respectively) than controls, whereas the survival rate of RCN-SS-TD-MoG recipients (78%) was marginally insignificant compared to controls. It is possible that larger numbers of mice or higher challenge doses may have provided clearer distinctions between vaccine constructs.

When developing a virus vector vaccine, primary considerations include stability, safety, infectivity, and the ability to express the target antigen at high levels. The synthetic early/late promoter (S E/L) was added to RCN-MoG to enhance transcription and, subsequently, protein expression, however, this promoter was designed and optimized for use in vaccina expression vectors [28], not RCN. In the RCN-tPA-MoG gene cassette, the S E/L promoter was replaced with the strong PrH5M promoter, as it is known to initiate higher levels of transcription and gene expression. The PrH5m promoter is a modified version of a native poxvirus promoter that increases stability of recombinant cassette insertion while retaining infectivity of the vector virus [19]. The success of the PrH5M promoter is indicated by the proliferation of antigen production in cells infected with RCN-tPA-MoG in the immunofluorescence assay.

The addition of the tPA signal sequence has been used in other gene expression systems to drive targeted protein production into the cellular secretion pathway and improve immunogenicity [29]. We added tPA to the second-generation construct to enhance protein expression and, thus, increase antibody production in vivo. In a study that assessed multiple recombinant raccoonpox vaccine candidates for sylvatic plague, the addition of tPA resulted in a 20-fold increase of target protein produced in vitro when compared to the same vaccine construct without tPA after 48 h of infection [30]. Another study assessed efficacies of two recombinant vaccinia vector vaccines against tuberculosis (one with tPA, one without) and found that mice that received the construct with the tPA secretory signal produced significantly higher levels of IgG [31]. Furthermore, the addition of tPA enhanced the cellular immune response in vaccinated mice in that study by increasing levels of measurable cytokines and T cells [31]. However, in other studies the use of a secretory signal appeared to boost protein expression in vitro but did not result in significantly higher antibody titers in mice [32], much like the outcome of our present study.

Although inoculation with RCN-SS-TD-MoG resulted in comparatively lower survival in vivo, serological analysis indicated that this construct produced quantities of antibodies comparable to the other vaccine constructs, which is consistent with the modifications made to the gene cassette. The S E/L promoter was retained in this construct to augment transcription and protein expression, as it did for RCN-MoG. The human IgG signal peptide was added to the cassette to enhance MoG expression. The addition of this signal peptide in a previous study resulted in a 1000-fold increase of G protein excreted in cell culture, and the recombinant G protein produced by the expressing cells induced antibody production in mice [33].

Despite the demonstrated effects of our genetic additions on immunogenicity, vaccination with RCN-SS-TD-MoG was marginally insignificant in the survival analysis, which may be due to a lack of statistical power. One mouse in this group died prior to challenge, which reduced the sample size for effective comparison. The two mice that succumbed to the challenge from this group should have theoretically been protected, as both had detectable RVNAs post vaccination and pre-challenge. However, results from previous rabies vaccine studies indicate that seroconversion post-vaccination may not directly correlate with survival, as measurable antibodies do not always reflect an accurate estimate of protective efficacy [34,35]. Furthermore, the human IgG signal peptide was previously used to generate high levels of G protein in HEK cells and later Chinese hamster ovary (CHO) cells [33]. HEK cells are often used for expression in biomedical settings because of their accuracy of translation and their high efficiency of transfection and protein production [36,37]. In contrast, we used Vero cells to grow our recombinant viruses and assess G expression, which may not have been the optimal cell line to maximize protein production and elicit potent antibodies in vivo. The deficiencies in immunogenicity and survivorship, as demonstrated in the mouse model, indicate that RCN-SS-TD-MoG may not be worth pursing further as a potential vaccine candidate.

Mice inoculated with RCN-tPA-MoG and RCN-MoG had survival rates that were largely consistent with our previous work, in which mice that received RCN-MoG had a 100% survival rate [14]. Although these data are promising, additional animal studies and experiments would be necessary to further determine the most efficacious candidate. Specifically, new mouse studies with higher vaccine doses or with larger sample sizes would be useful to generate more robust survival data and further elucidate the immunogenicity of each construct. In vitro expression would need to be further validated through additional methods of quantification. Western blots can be quantified by using protein loading controls such as beta actin or GADPH to evaluate quantities of protein in an SDS-PAGE gel. Beta actin and GADPF are “housekeeping genes” that are highly conserved in mammalian cells; incubation with anti-beta actin or anti-GADPH antibodies can be used to compare quantities of target proteins in western blot assays and other types of protein analysis [38,39].

In a recent study, RCN-MoG was tested in vampire bats by both oral and topical delivery routes. Although serology results were inconclusive, vaccinated bats that succumbed to rabies challenge did not shed RABV in their saliva, indicating that vaccination with RCN MoG may disrupt transmission [40]. RCN-tPA-MoG may have a similar effect on inhibiting viral shedding and inducing antibody production in the target species, but both constructs would need to be tested comparatively and through oral delivery in a captive animal study to confirm. The results from our current study indicate that further improvements to an oral rabies vaccine targeting bats could lead to the development of an efficacious tool for reducing rabies disease burden in free ranging bat populations, and, subsequently, reduce the potential for human and animal exposure. Previous work in other terrestrial carnivore species demonstrates that oral vaccination is a feasible method of inducing protective immunity in wildlife, but additional studies would be useful to determine which vaccine candidate will be most effective in bats.

## 5. Conclusions

In conclusion, we determined how and if molecular modifications improved the efficacy of RCN-vectored vaccines by assessing MoG expression in vitro, RVNA production in vivo, and survival in the mouse model after challenge with RABV. Furthermore, we demonstrated that vaccination with either RCN-tPA-MoG or RCN-MoG provides sufficient protection from rabies infection in mice. Our data, in combination with previous work, indicates that RCN-MoG and RCN-tPA-MoG could be further evaluated and may be effective vaccine candidates to protect bats against rabies.

## Figures and Tables

**Table 1:** Mean of fluorescent particles expressing MoG (with standard deviation), counted from images of immunofluorescence assays. RCN-tPA-MoG has the highest mean, followed by RCN-SS-TD-MoG and RCN-MoG; a significant difference was detected between the three viruses (*p* = 0.004).

Virus	RCN-MoG	RCN-tPA-MoG	RCN-SS-TD-MoG
Mean fluorescent particles	144.0	2295.7	1488.3
SD	48.383	104.222	260.776

## Data Availability

All data collected for this study are available at https://www.sciencebase.gov/catalog/item/6196c135d34eb622f691acc6 (accessed on 2 December 2021) [41].
